# Ecology and Biodiversity Ontology Alignment for Smart Environment *via* Adaptive Compact Evolutionary Algorithm

**DOI:** 10.3389/fpls.2022.877120

**Published:** 2022-04-14

**Authors:** Xingsi Xue, Pei-Wei Tsai

**Affiliations:** ^1^Fujian Provincial Key Laboratory of Big Data Mining and Applications, Fujian University of Technology, Fuzhou, China; ^2^Intelligent Information Processing Research Center, Fujian University of Technology, Fuzhou, China; ^3^Department of Computer Science and Software Engineering, Swinburne University of Technology, Hawthorn, VIC, Australia

**Keywords:** ecology ontology, biodiversity ontology, ontology alignment, adaptive compact evolutionary algorithm, semantic reasoning

## Abstract

Smart Environment (SE) focuses on the initiatives for healthy living, where ecological issues and biodiversity play a vital role in the environment and sustainability. To manage the knowledge on ecology and biodiversity and preserve the ecosystem and biodiversity simultaneously, it is necessary to align the data entities in different ecology and biodiversity ontologies. Since the problem of Ecology and Biodiversity Ontology Alignment (EBOA) is a large-scale optimization problem with sparse solutions, finding high-quality EBOA is an open challenge. Evolutionary Algorithm (EA) is a state-of-the-art technique in the ontology aligning domain, and this study further proposes an Adaptive Compact EA (ACEA) to address the problem of EBOA, which uses semantic reasoning to reduce searching space and adaptively guides searching direction to improve the algorithm's performance. In addition, we formally model the problem of EBOA as a discrete optimization problem, which maximizes the alignment's completeness and correctness through determining an optimal entity corresponding set. After that, a hybrid entity similarity measure is presented to distinguish the heterogeneous data entities, and an ACEA-based aligning technique is proposed. The experiment uses the famous Biodiversity and Ecology track to test ACEA's performance, and the experimental results show that ACEA-based aligning technique statistically outperforms other EA-based and state-of-the-art aligning techniques.

## 1. Introduction

Smart Environment (SE) is a knowledge-based system that focuses on initiatives for healthy living with an emphasis on environment and sustainability, where ecological issues and biodiversity play a vital role in urban citizens' life (Kumar, 2020). In particular, ecology research focuses on the ecosystems, habitat restoration practices, and communities of interest, which is of help to further our understanding of the environment and opportunities to affect change; biodiversity investigates the species' variability as well as their relationship to the environment. Since ecology and biodiversity are the most complex entities on this planet, the corresponding knowledge is usually modeled with the ontology (Madin et al., 2008), which is a powerful domain knowledge modeling technique (Berners-Lee et al., 2001). Currently, more and more ecology and biodiversity ontologies have been developed, such as Environment Ontology (ENVO) and Plant Trait Ontology (PTO). However, since they are developed and maintained independently, a concept might be defined with different contexts, granularities, and terminologies, yielding the ontology heterogeneity problem (Karam et al., 2020). Examples of heterogeneous ecology and biodiversity ontologies are shown in Table 1.

**Table 1:** The examples of heterogeneous ecology and biodiversity ontologies.

**ENVO ontology^**a**^**	**SWEET ontology^**b**^**
Divergent tectonic movement	Plate divergence
Tectonic movement	Continental drift
**FLOTO ontology^**c**^**	**PTO ontology^**d**^**
Inflorescence absent	Inflorescenceless
Leaf alternate placement	Phyllotaxy

a *http://agroportal.lirmm.fr/ontologies/ENVO*.

b *https://bioportal.bioontology.org/ontologies/SWEET*.

c *http://agroportal.lirmm.fr/ontologies/FLOPO*.

d *http://agroportal.lirmm.fr/ontologies/TO*.

Therefore, to preserve the ecosystem and biodiversity simultaneously and manage the knowledge on ecology and biodiversity, it is necessary to link the data entities in different ecology and biodiversity ontologies, which is the so-called Ecology and Biodiversity Ontology Alignment (EBOA).

Aligning ecology and biodiversity ontologies aims at finding a 0–1 Aligning Matrix (AM), whose element denotes whether two corresponding entities (the source ontology's entities in row and the target ontology's entities in column) are mapped by 1 or not by 0. Since the scale of the ecology and biodiversity ontologies are usually large, and the constraint of single cardinality on the aligning result, the problem of EBOA needs to find a large-scale AM (the number of its row and column is large) with sparse solutions (most of its element values are 0). Due to the large search space and richness of semantic meaning on different data entities, it is a complex task of aligning ecology and biodiversity ontologies. In recent years, Evolutionary Algorithm (EA) (Mirjalili, 2019) has become a popular technique in the ontology aligning domain (Acampora et al., 2013; Xue et al., 2018). Due to the population-based evolving paradigm, the classic EA's searching performance is low in terms of memory consumption and run time. To improve the efficiency, a new category of EA with the name Compact EA (CEA) is presented, which uses compact encoding mechanism to describe the whole population with probability estimation. CEA mimics EA's searching process by simplifying the evolving operators, but it is easy to get stuck in the local optima especially when two ontologies' scale is large. To overcome this drawback, this study further proposes an Adaptive CEA (ACEA), which uses the semantic reasoning to filter the negative correspondences, and adaptively alters the algorithm's searching direction to explore the unknown region. In the following, we list the contributions of this study:

- The optimization model of the problem of EBOA is presented;
- A hybrid entity similarity measure is proposed to distinguish the heterogeneous ecology and biodiversity data entities;
- An ACEA-based aligning technique is proposed, which uses semantic reasoning to reduce searching space, and adaptively guides the searching direction to efficiently align the ecology and biodiversity ontologies.

The introduction process of this study is as follows: before defining the problem of EBOA and entity similarity measure (Section 3), the EA-based aligning techniques are overviewed (Section 2); after that, the problem-specific ACEA is presented (Section 4), followed by the experimental results (Section 5); and finally, we draw the conclusion on this article's study (Section 6).

## 2. Evolutionary Ontology Aligning Technique

With the rapid development of ontology engineering, the scale of an ontology has grown from hundreds of entities to tens of thousands of entities, and the semantic representation of the entities also become more and more complex, which makes the determination of a high-quality ontology alignment become an open challenge (Shvaiko and Euzenat, 2011). Essentially, the ontology aligning problem can be regarded as an optimizing issue that aims at maximizing the quality of final alignment, and EA-based aligning techniques have become a popular methodology to address this problem.

The first EA-based ontology aligning technique is proposed by Martinez-Gil et al. (2008) which tries to find an optimal way of combining different similarity measures for determining the final alignment. Later on, researchers have done a lot to improve this category of EA-based aligning techniques. Based on this study, Ginsca and Iftene (2010) further optimize the threshold for filtering final alignment. Acampora et al. (2012) propose a Hybrid EA (HEA) to improve the efficiency of classic EA's performance. Alves et al. (2012) further use the instance-level information in an ontology to construct the similarity measure and then use HEA to combine it with others. Currently, it is necessary to enhance the performance of population-based EA in terms of running time and memory so as to address the large-scale aligning task, such as addressing the problem of EBOA where the ontology contains tens of thousands of entities. To this end, an efficiency improvement strategy should be introduced to improve classic EA's performance. The most popular way is the utilization of a compact encoding based evolving paradigm, which describes the population with a probability distribution, and on this basis, it approximates the classic EA's evolving process. The first CEA-based aligning technique is proposed in Xue et al. (2015), which executes the evolving process by one Probability Vector (PV). According to the experimental results, CEA is able to significantly reduce EA's running time and memory consumption without sacrificing the alignment's quality. Later on, a Hybrid CEA (HCEA) (Xue and Wang, 2015a) and a CEA with a Re-sample Inheritance Mechanism (RIM) (Xue and Liu, 2022) are respectively proposed to further enhance CEA's performance. To address the large-scale aligning task, a divide-and-conquer method is also presented, which is of help to reduce HCEA's searching space (Xue and Wang, 2015b; Xue and Zhang, 2021).

Existing EA-based aligning approaches need to maintain each similarity measure's corresponding AM, and on this basis, the optimization on the alignment can be executed, which greatly raises the computational complexity. In this study, we try to directly find a set of correspondences with the given similarity measure, which only needs to save several entity pairs' similarity value instead of maintaining all the similarity measures' corresponding entity pairs' similarity values. In addition, classic CEA only uses one PV to execute the optimizing process, which makes it easy to get stuck in the local optima when facing a complex optimization problem. To overcome this drawback, our approach proposes to adaptively maintain several Probability Matrices (PMs) to guide the algorithm's searching direction. Finally, since the problem of EBOA is a large-scale issue with sparse solutions, we propose semantic reasoning based initialization to reduce the algorithm's searching space and evenly distribute the AM's element values.

## 3. Ecology and Biodiversity Ontology Alignment

### 3.1. The Problem of Ecology and Biodiversity Ontology Alignment

**Figure 1:**
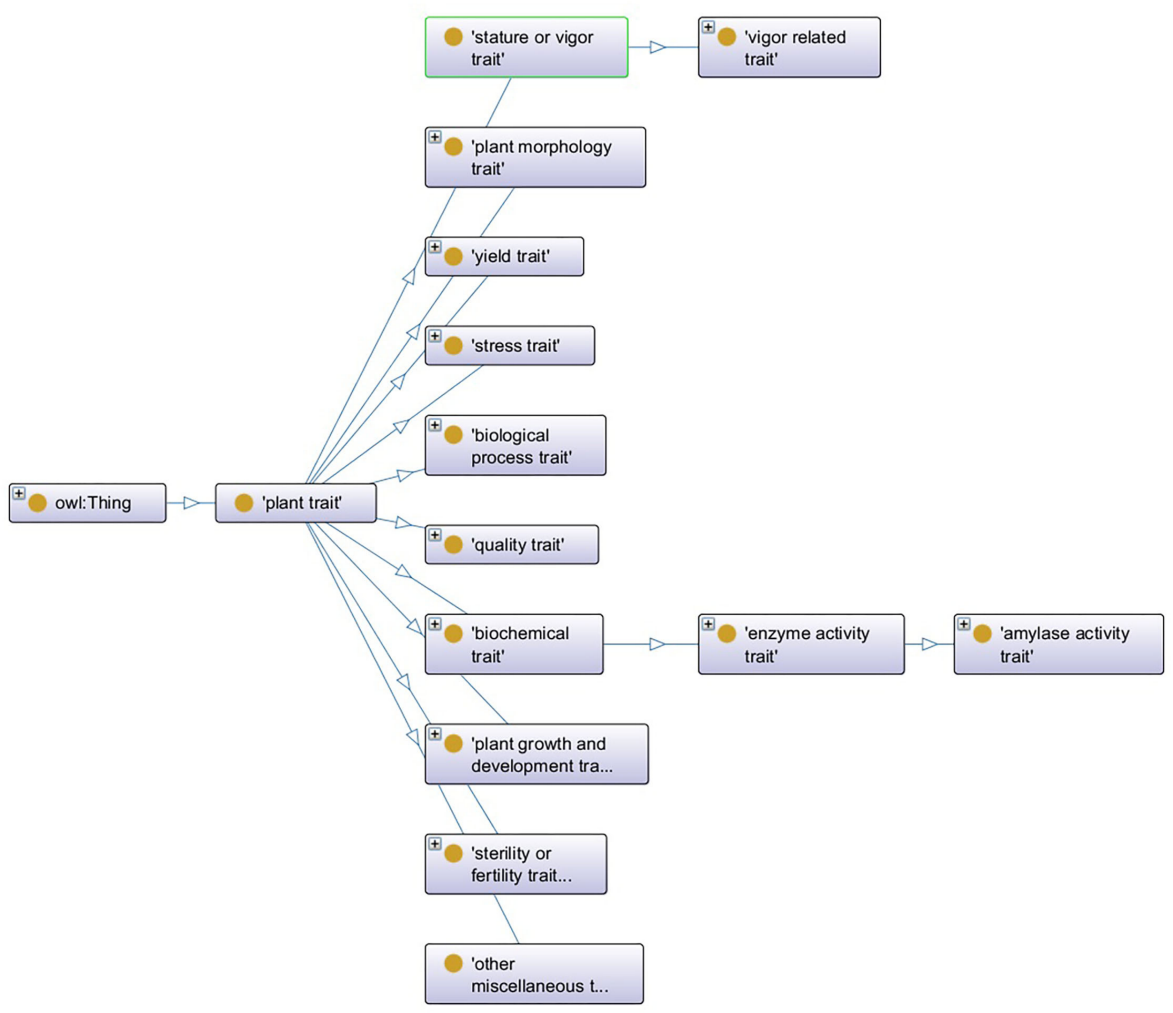
A segment of plant trait ontology.

An ontology consists of the concepts, the datatype properties, and the object properties, which are referred to as entities (Xue et al., 2021). Figure 1 shows a segment of PTO, where the oval symbol describes the concept's name, e.g., “plant trait,” the arrow line is the object property or relationship between two concepts, e.g., the concept “quantity trait” is subsumed by “plant trait,” and each concept has several datatype properties to describe its feature, e.g., the concept “plant trait” has the datatype property “definition” whose value is “A plant trait (TO:0000387) that is the commercial and /or economical value of the plant product, or its overall improvement.”

An entity correspondence consists of 4 elements, i.e., *e*_1_, *e*_2_, *rel, conf*, where *e*_1_ and *e*_2_ are respectively two ontologies' entities, *rel* is the type of their relationship (typically the equivalence ≡), and *conf* denotes the confidence level that the correspondence holds, which is often measured by *e*_1_ and *e*_2_'s similarity value. For example, in Table 1, (*ENVO*:*Tectonicmovement, SWEET*:*Continentaldrift*, ≡, 0.9) denote a correspondence between the concept “Tectonic movement” from ENVO and the concept “Continental drift” from SWEET, their relationship is the equivalence ≡, and this correspondence's confidence value is 0.9. The ontology alignment is a correspondence set, whose quality is typically evaluated with recall, precision, and f-measure (Rijsberge, 1975). Since reference alignment is often not available in the practical aligning tasks, this study proposes three new metrics to approximate them. According to Wang et al. (2006), *recall*(*A*) can be estimated by the number of correspondences found in *A*, i.e., *recall*′(*A*) = *norm*(|*A*|), *precision*(*A*) can be evaluated by the average similarity values of all the correspondences in *A*, i.e., $recall^{\prime }\left(A\right)=\frac{\sum sim\left(corr_{i}\right)}{\left|A\right|}$ where *corr*_*i*_ is *i*-th correspondence in *A*. To evaluate an alignment's quality in terms of both completeness and correctness, a comprehensive metric $f-measure^{\prime }\left(A\right)=\frac{2\times recall^{\prime }\left(A\right)\times precision^{\prime }\left(A\right)}{recall^{\prime }\left(A\right)+precision^{\prime }\left(A\right)}$ is presented, which calculate an alignment's harmony mean of its *recall*′(*A*) and *precision*′(*A*). Given two ontologies *O*_1_ and *O*_2_, a 0–1 matrix *M* and its corresponding alignment *A*, the problem of EBOA is defined as follows:


(1) $$\{\begin{array}{ll} max & f(M)\\ s.t. & M={\left[m_{i,j}\right]}_{\left|O_{1}|\times |O_{2}\right|}\\ & m_{i,j}\in \{0,1\},i=1,2,\cdots ,|O_{1}| \end{array}$$

where |*O*_1_| and *O*_2_ are respectively *O*_1_ and *O*_2_'s entity numbers, and *f*(*M*) is equal to *f*−*measure*′(*A*), and the model of EBOA aims at finding an optimal matrix by maximizing its corresponding alignment's *f*−*measure*′. In particular, the decision variable is a 0–1 matrix whose row and column are respectively two ontologies' entities, and its element value 1 means two corresponding entities are mapped, and 0 means not.

### 3.2. Entity Similarity Measure

The entity similarity measure calculates two entities' similarity value *conf*, which is a real number in [0,1]. *conf* = 1 means two entities are the same, and *conf* = 0 means they are totally different. To improve the result's confidence, usually, it is necessary to comprehensively consider three categories of similarity measures, i.e., string-based, linguistic-based, and structure-based similarity measures (Xue and Huang, 2022). To this end, this study proposes a hybrid entity similarity measure to comprehensively calculate the similarity value: (1) given two entities *e*_1_ and *e*_2_, before calculating their similarity value, the numbers, punctuations, and stop-words in their names are first removed; (2) the strings are split into the words, which are further lemmatized and stemmed; (3) in each word set, the word will be removed if it is the same literally or synonymous to the other one in Wordnet (Miller, 1995), and we obtain two word sets *s*_1_ and *s*_2_; finally, *e*_1_ and *e*_2_'s similarity value is the same as the similarity value of two string *s*_1_ and *s*_2_:


(2) $$\begin{array}{r} sim\left(s_{1},s_{2}\right)\\ =\frac{\overset{\left|W_{1}\right|}{\underset{i=1}{\sum }}\underset{j=1\cdots |W_{2}|}{\max}\left(sim\left(w_{1,i},w_{2,j}\right)\right)+\overset{\left|W_{2}\right|}{\underset{j=1}{\sum }}\underset{i=1\cdots |W_{1}|}{\max}\left(sim\left(w_{1,i},w_{2,j}\right)\right)}{\left|W_{1}|+|W_{2}\right|} \end{array}$$

where |*W*_1_| and |*W*_1_| are respectively the numbers of words in *W*_1_ and *W*_2_, and *w*_1, *i*_ and *w*_2, *j*_ are respectively the *i*th and *j*th words in *W*_1_ and *W*_2_; and *sim*(*w*_1, *i*_, *w*_2, *j*_) is calculated with Wordnet and N-gram measure (Kondrak, 2005):


(3) $$sim(w_{1,i},w_{2,j})=\{\begin{array}{ll} 1, & \text{two words are synonyms}\\ & \text{in Wordnet}\\ N\text{-}gram(w_{1,i},w_{2,j}), & \text{otherwise} \end{array}$$

## 4. Adaptive Compact Evolutionary Algorithm

Adaptive compact evolutionary algorithm adaptively maintains PMs according to the current generation's population information, which is able to help the algorithm effectively exploit the unexplored domains. In addition, ACEA uses the anchor-based semantic reasoning strategy to initialize the individual and refine the new individuals, which can effectively reduce the algorithm's searching domain. The framework of ACEA is presented in Algorithm 1, which takes as input two ontologies to be aligned, and the output the alignment with best fitness value.

**Table:** **Algorithm 1** The Framework of Adaptive Evolutionary Algorithm

*PM*_*num*_=2; //Initialize the number of PM
**for** *i* = 0; *i*<*PM*_*num*_; *i*++ **do**
$AM_{elite}^{i}=initializeAM\left(PM^{i}\right)$; //Initialize the elite AM
**end for**
*gen* = 0;
**while** *gen*<*maxGen* **do**
for *i* = 0; *i*<*PM*_*num*_; *i*++ **do**
*Update*(*PM*^*i*^); //Execute the evolutionary operator and update PM
**end for**
*PM*_*num*_ = *adaptivePMMaintenance*(); //Adaptively maintain the PMs
*gen* = *gen*+1;
**end while**

In the next, we successively present the Semantic Reasoning Based Initialization and adaptive PM maintenance.

### 4.1. Semantic Reasoning Based Initialization

**Figure 2:**
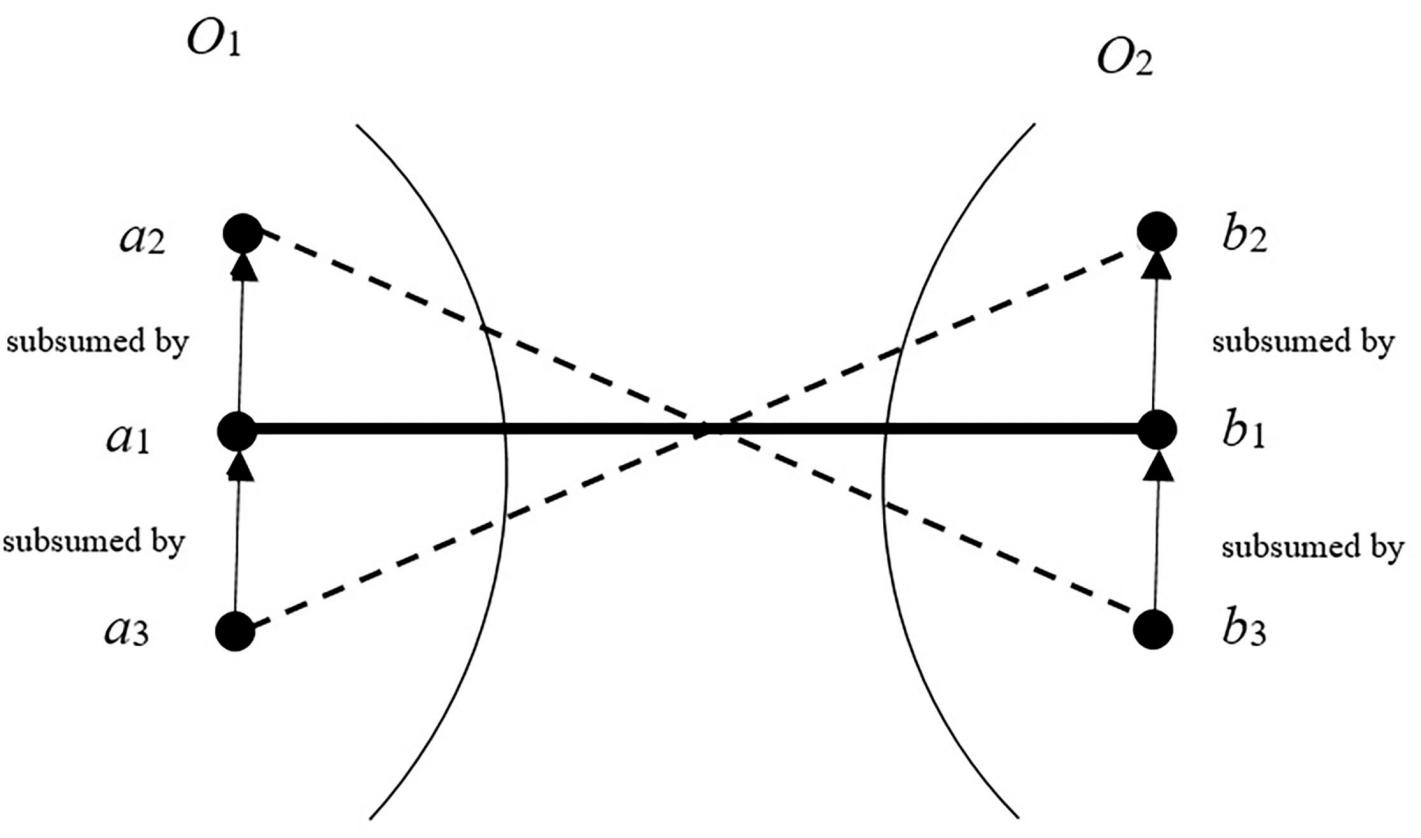
Logical contradiction between correspondences.

Typically, the correspondence with a high confidence value is referred to as Positive Anchor (PA), and the one with a low confidence value is called the Negative Anchor (NA) (Wang, 2010). The concepts in an ontology are modeled with the hierarchy graph (Chu et al., 2020), where the node denotes the concept and the edge represents the relationships between two concepts. Figure 2 shows an example of correspondences' logical contradiction. As shown in the figure, the entities *a*_1_, *a*_2_, and *a*_3_ are three entities of ontology *O*_1_, and the entities *b*_1_, *b*_2_, and *b*_3_ belong to ontology *O*_2_. In *O*_1_ (*O*_2_), *a*_3_ (*b*_3_) is subsumed by *a*_1_ (*b*_1_), and *a*_1_ (*b*_1_) is subsumed by *a*_2_ (*b*_2_). Assuming the correspondence (*a*_1_, *b*_1_) is a PA, the correspondences (*a*_2_, *b*_3_) and (*a*_3_, *b*_2_) logically contradict with (*a*_1_, *b*_1_). It is obvious that the correspondences that contradict with some PA will not hold, and the confidence of correspondences that are in line with some NA should be reduced. According to this reasoning rule, the searching space of the algorithm can be reduced.

**Figure 3:**
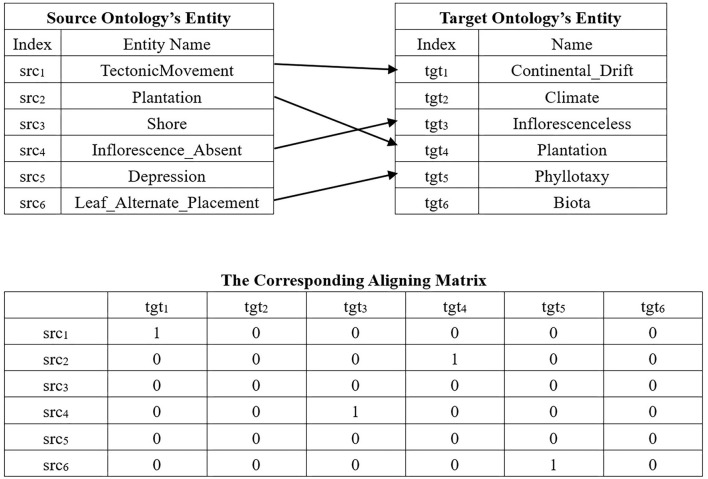
An example of encoding mechanism.

Each individual of ACEA is represented by a 0–1 matrix, i.e., the so-called AM. Figure 3 shows an example of an encoding mechanism, wherein the top of the figure is a real alignment, and its corresponding AM is given below it. ACEA uses Probability Matrix (PM) to approximately describe the population, which has the same size as AM. PM's elements are the real number in [0,1], which denotes the probability of being 1 with respect to the corresponding gene bit. Therefore, we can use PM to generate AM by comparing its elements with a random number in [0,1].

Since the problem of EBOA is a large-scale issue with sparse solutions, it is necessary to evenly distribute the gene value when initializing AM. Algorithm 2 shows the pseudo-code of initialization.

**Table:** **Algorithm 2** Initialization

Initialize Aligning Matrix *AM* by setting all its elements as 0;
**if** Probability Matrix *PM* is not given **then**
initialize all the elements in *PM* as 0.5;
**end if**
initialize the Positive Anchor Set *PAS*;
**for** *i* = 0; *i*<*PAS*.*length*; *i*++ **do**
for *j* = 0; *j*<*AM*.*row*; *j*++ **do**
for *k* = 0; *k*<*AM*.*column*; *k*++ **do**
if (*entity*_*i*_, *entity*_*j*_) is contradicted with *PAS*_*i*_ **then**
*PM*_*i, j*_ = 0;
**else**
if *random*(0, 1) < *PM*_*i, j*_ **then**
*AM*_*i, j*_ = 1;
**end if**
**end if**
**end for**
**end for**
**end for**

We first initialize *AM* by setting all its elements as 0 and determine the positive anchor set *PAS* with the similarity measure. If probability matrix *PM* is not given, all its elements will be initialized as 0.5. Then, we compare all the correspondences in AM with *PAS*. If the correspondence is logically conflicted with *PAS*'s correspondence, its *AM* and *PM*'s values will be set as 0, otherwise, its value is decided by comparing its corresponding *PM*'s value with a random number in [0,1]. Through semantic reasoning, the searching space can be significantly reduced, and initializing *PM*'s elements as 0.5 is also of help to ensure the even distribution of the gene values.

### 4.2. Updating Probability Matrix

Adaptive compact evolutionary algorithm combines the mechanisms of a classic EA with a competitive learning mechanism, which is effective to lead the algorithm to determine the optimal solution. To be specific, ACEA first uses its fitness function to evaluate its solution's fitness value by calculating its corresponding alignment's *f*−*measure*′, and through competitions between the individuals, the algorithm updates PM by moving it toward the elite individual. The process of updating PM is presented in Algorithm 3.

**Table:** **Algorithm 3** Updating Probability Matrix

*AM*^*new*^ = *PM*.*generateAM*();
*AM*′ = *PM*.*generateAM*();
**for** *i* = 0; *i*<*AM*^*new*^.*row*; *i*++ **do**
for *j* = 0; *j*<*AM*^*new*^.*column*; *j*++ **do**
if *random*(0, 1) <0.5 **then**
$AM_{i,j}^{new}=AM_{i,j}^{\prime }$;
**end if**
**end for**
**end for**
*compete*(*AM*^*new*^, *AM*^*elite*^);
**if** *winner* = =*AM*^*new*^ **then**
*AM*^*elite*^ = *AM*^*new*^;
**end if**
**for** *i* = 0; *i*<*PM*.*row*; *i*++ **do**
for *j* = 0; *j*<*PM*.*column*; *j*++ **do**
if $AM_{i,j}^{elite}==1$ **then**
$PM_{i,j}^{elite}+=0.01$;
**else**
$PM_{i,j}^{elite}-=0.01$;
**end if**
**end for**
**end for**
**for** *i* = 0; *i*<*PM*^*elite*^.*row*; *i*++ **do**
for *j* = 0; *j*<*PM*^*elite*^.*column*; *j*++ **do**
if *corr*(*e*_*i*_, *e*_*j*_).*conf* <0.2 **then**
$PM_{i,j}^{elite}=0$;
if *corr*(*e*_*m*_, *e*_*n*_) is *corr*(*e*_*i*_, *e*_*j*_)'s neighbor correspondence **then**
if *corr*(*e*_*m*_, *e*_*n*_) does not logically contradict with *corr*(*e*_*i*_, *e*_*j*_) **then**
$PM_{m,n}^{elite}-=0.01$;
**end if**
**end if**
**end if**
**end for**
**end for**

In Algorithm 3, we first generate two AMs and use them to obtain a new AM *AM*^*new*^ with the uniform crossover operator. Then, *AM*^*new*^ is compared with the elite AM *AM*^*elite*^, and the winner will become the elite AM. After that, we use *AM*^*elite*^ to update its corresponding PM: if $AM_{i,j}^{elite}==1$, then $PM_{i,j}^{elite}+=0.01$; otherwise, $PM_{i,j}^{elite}-=0.01$. We update PM so that the newly generated AM will be closer to the elite AM. Finally, we find the NA *corr*(*e*_*i*_, *e*_*j*_) from *AM*^*elite*^, and their neighbor correspondence *corr*(*e*_*m*_, *e*_*n*_) where the shortest path between *e*_*m*_ (or *e*_*n*_) and *e*_*i*_ (or *e*_*j*_) in the ontology hierarchy graph is less than 2, we pick up those do logically contradict with *corr*(*e*_*i*_, *e*_*j*_) and reduce their corresponding *PM* elements' values by 0.01. In particular, the step length of updating *PM* determines the algorithm's learning rate. If the step length is too large, the algorithm converges quickly, i.e., the value of *PM*‘s elements are close to 1 or 0; and if it is too small, the algorithm consumes a long running time. Here, we empirically set the step length as 0.01, which is able to ensure the highest average quality of alignments on all testing cases.

### 4.3. Adaptive Probability Matrix Maintenance

At the end of the generation, adaptive PM maintenance is executed to adjust the algorithm's searching direction. The pseudo-code of adaptive population maintenance is shown in Algorithm 4.

**Table:** **Algorithm 4** Adaptive Probability Matrix Maintenance

**if** $\sum \left(\left|PM_{i,j}^{a}-PM_{i,j}^{b}\right|\right)<0.5$ **then**
if $PM_{elite}^{a}$ is better than $PM_{elite}^{b}$ **then**
remove *PM*^*b*^;
**else**
remove *PM*^*a*^;
**end if**
**end if**
**if** All elite AMs keep unchanged for δ generations **then**
for *i* = 0; *i*<*PM*^*new*^.*row*; *i*++ **do**
for *j* = 0; *j*<*PM*^*new*^.*column*; *j*++ **do**
if $PM_{i,j}^{max}=PM_{i,j}^{min}=1or0$ **then**
$PM_{i,j}^{new}=1or0$;
**end if**
**if** $PM_{i,j}^{max}<0.5$ **then**
$PM_{i,j}^{new}=PM_{i,j}^{max}+rand\left(0,1\right)\left(1-PM_{i,j}^{max}\right)$;
**end if**
**if** $PM_{i,j}^{min}>0.5$ **then**
$PM_{i,j}^{new}=\left(1-rand\left(0,1\right)\right)PM_{i,j}^{min}$;
**end if**
**if** $PM_{i,j}^{max}>0.5$ and $PM_{i,j}^{min}<0.5$ **then**
$PM_{i,j}^{new}=0.5$;
**end if**
**end for**
**end for**
$AM_{elite}^{new}=initializeAM\left(PM^{new}\right)$;
**end if**

In Algorithm 4, we first calculate the distance between the existing PMs. The smaller distance indicates a larger overlap between their searching directions, and therefore, the one with worse elite AM should be deleted. When all elite AMs keep unchanged for δ generations, i.e., the algorithm gets stuck in the local optima, we add a new PM *PM*^*new*^ with a different searching direction. To determine *PM*^*new*^'s elements, we need to analyze the existing PMs' elements distribution through the maximum probability and minimum probability. To be specific, for each of *PM*^*new*^'s element $PM_{i,j}^{new}$, we find its corresponding maximum probability $PM_{i,j}^{max}$ and minimum probability $PM_{i,j}^{min}$ from the existing PMs. If they are all equal to 1 or 0, we will set $PM_{i,j}^{new}$ as 1 or 0. If $PM_{i,j}^{max}<0.5$, $PM_{i,j}^{new}$ will be put in the left of all existing probabilities, i.e., $PM_{i,j}^{new}=PM_{i,j}^{max}+rand\left(0,1\right)\left(1-PM_{i,j}^{max}\right)$. If $PM_{i,j}^{min}>0.5$, $PM_{i,j}^{new}$ will be put in the right of all existing probabilities, i.e., $PM_{i,j}^{new}=\left(1-rand\left(0,1\right)\right)PM_{i,j}^{min})$. If $PM_{i,j}^{max}>0.5$ and $PM_{i,j}^{min}<0.5$, $PM_{i,j}^{new}$ will be put in the middle, i.e., $PM_{i,j}^{new}=0.5$. Finally, we initialize the elite AM $AM_{elite}^{new}$ for *PM*^*new*^.

## 5. Experiment

### 5.1. Experimental Configuration

We use the track of Biodiversity and Ecology in Ontology Alignment Evaluation Initiative (OAEI)^1^ to test ACEA's performance. Biodiversity and Ecology track consists of four pairs of ontologies in the biodiversity and ecology domain: (1) ENVO^2^-SWEET^3^, (2) PTO^4^-FLOPO^5^, (3) AGROVOC^6^-NALT^7^, (4) GEMET^8^-ANAEETHES^9^. All of these ontologies are widely used in various projects and researches on biodiversity and ecology, which are developed in parallel and are significantly overlapping.

In the experiment, we compare ACEA with CEA (Xue et al., 2015), HCEA (Xue and Chen, 2019), and OAEI's participants. In particular, CEA's configuration is as follows:

- The maximum generation = 3,000;
- The step length for updating PV = 0.01.

The configuration of HCEA's is as following:

- The maximum generation = 3,000;
- The step length for updating PV = 0.01;
- The crossover probability = 0.6;
- The mutation probability = 0.03;
- The mutation shift = 0.05.

Additionally, ACEA's configuration is given as follows:

- The maximum generation = 3,000;
- The threshold for activate PM Maintenance = 60;
- The step length for updating probability matrix = 0.01.

Ontology Alignment Evaluation Initiative's participants' results are from OAEI's official website^10^. We first show the sensitivity testing on ACEA's parameter, then ACEA is compared with CEA and HCEA in terms of f-measure and convergence graph, and finally, ACEA is compared with the state-of-the-art ontology aligning techniques. ACEA, CEA, and HCEA's results are the mean value of 30 independent runs.

### 5.2. Experimental Results

**Figure 4:**
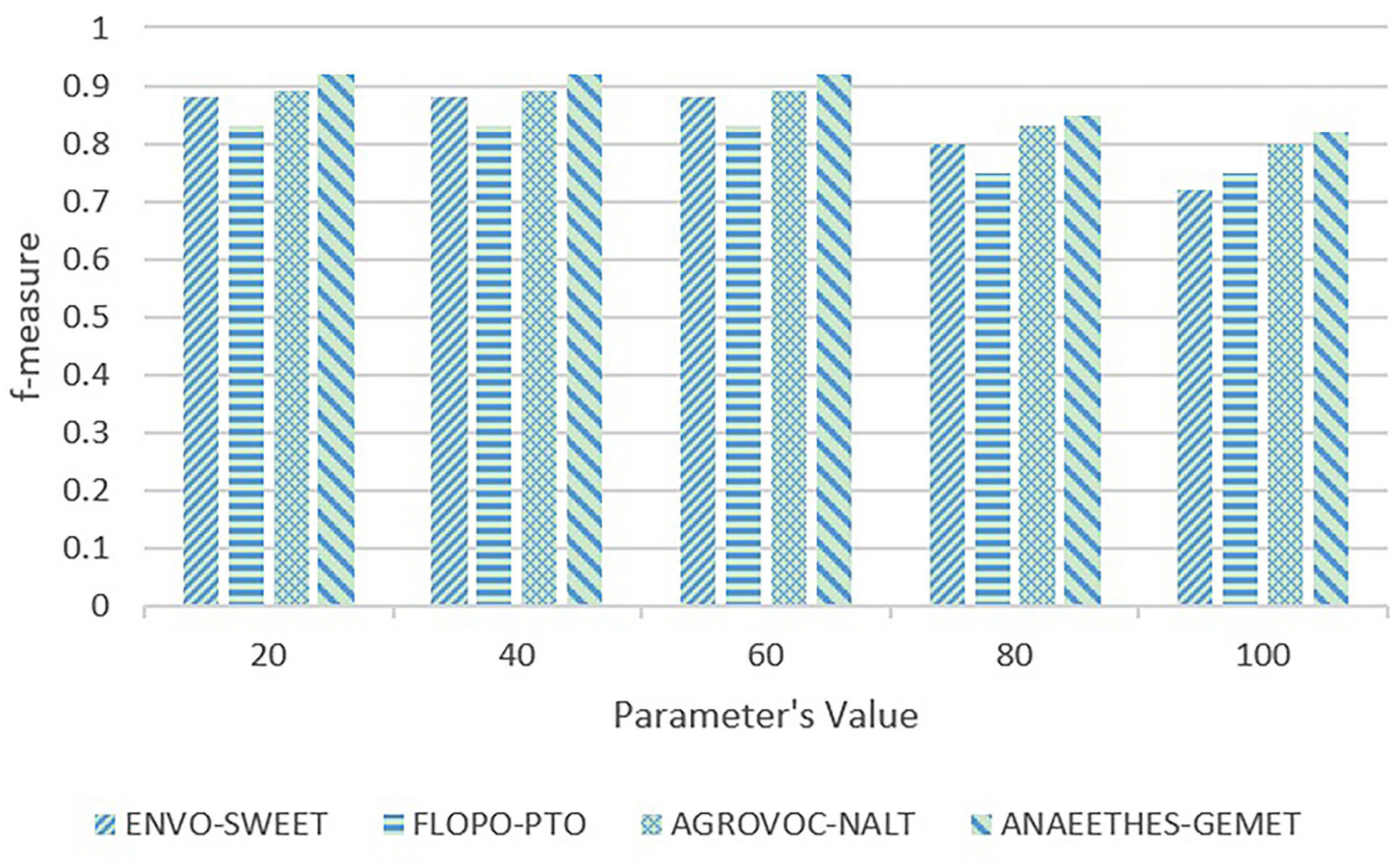
Sensitivity testing on Adaptive Compact EA's (ACEA) parameter δ in terms of f-measure.

**Figure 5:**
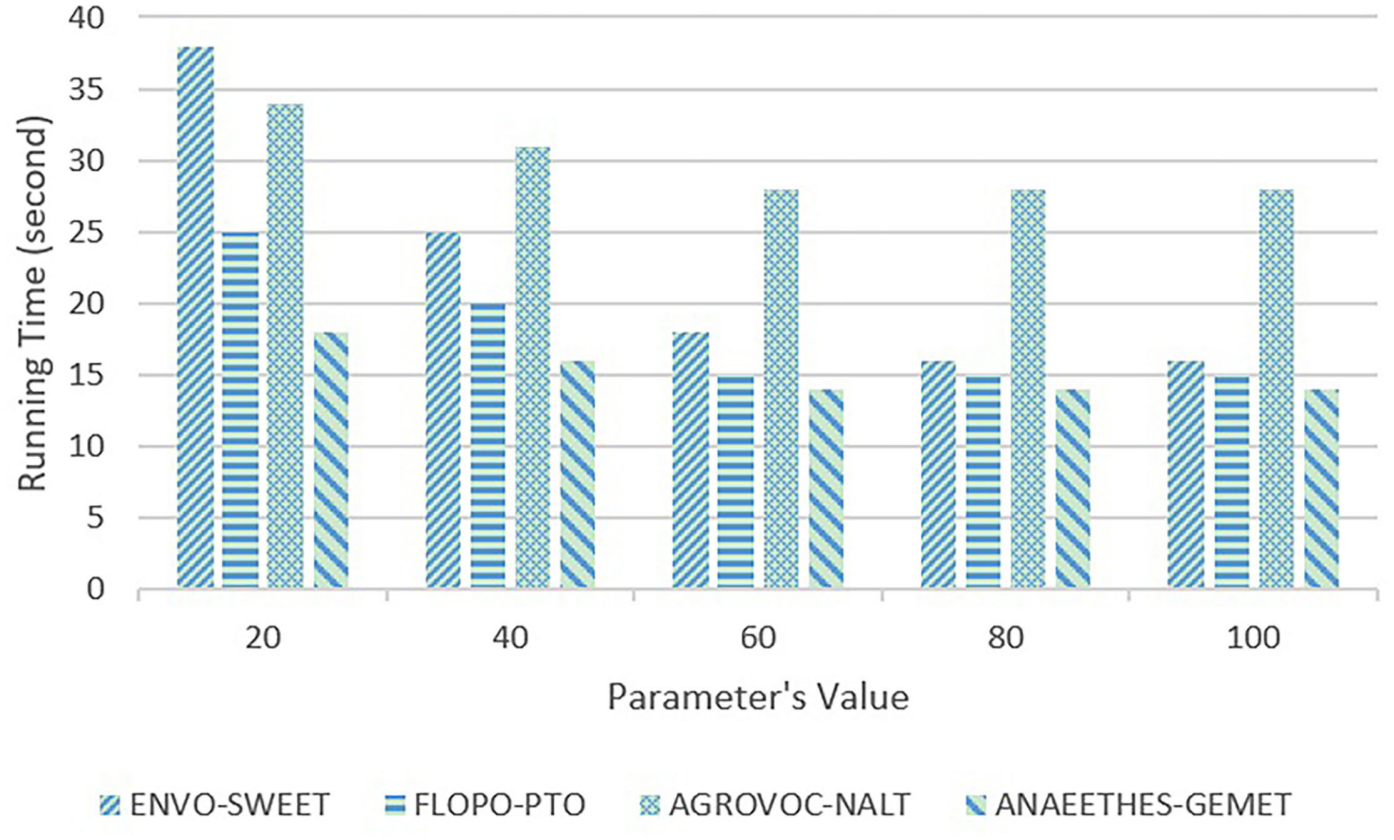
Sensitivity testing on ACEA's parameter δ in terms of running time (second).

First, the sensitivity testings are carried out on ACEA's parameter δ that determines the timing of executing the PM maintenance. If δ is too large, ACEA would get stuck in the local optima for a long time, which would hamper the algorithm from converging on the global optima, and if δ is too small, there would be too many PVs, which increases the computational complexity. We empirically take five representative values, i.e., 20, 40, 60, 80, and 100, to execute the sensitivity testing on δ, whose results are shown in Figures 4, 5.

In Figures 4, 5, with the increasing values, the quality of alignments begins to deteriorate when δ>60, and the running time start to decrease, and when δ = 60, it reaches the bottom. Therefore, the parameter δ = 60 is able to better trade-off the quality of alignments and the algorithm's running time.

In Table 2, we compare ACEA with CEA and HCEA with mean f-measure *f*-*measure* and the standard deviation *stdDev*. In Table 2, the statistical *t*-test (Schmetterer and Lehmann, 1962) is executed on the data presented in Table 3.

**Table 2:** Comparisons among Adaptive Compact EA (ACEA), Compact EA (CEA), and Hybrid CEA (HCEA) in terms of mean f-measure and standard deviation.

**Testing case**	**CEA**	**HCEA**	**ACEA**
	**f-measure**	**f-measure**	**f-measure**
	**(stdDev)**	**(stdDev)**	**(stdDev)**
ENVO-SWEET	0.79 (0.03)	0.83 (0.01)	0.88 (0.01)
FLOPO-PTO	0.76 (0.01)	0.76 (0.02)	0.83 (0.01)
AGROVOC-NALT	0.78 (0.03)	0.80 (0.01)	0.89 (0.02)
ANAEETHES-GEMET	0.74 (0.02)	0.82 (0.01)	0.92 (0.01)

**Table 3:** *T*-test on alignment's quality.

**Testing case**	**(CEA, ACEA)**	**(HCEA, ACEA)**
	***t*-value (*p*-value)**	***t*-value (*p*-value)**
ENVO-SWEET	–5.69 (0.014)	–7.07 (0.009)
FLOPO-PTO	–9.89 (0.005)	–6.26 (0.012)
AGROVOC-NALT	–6.10 (0.012)	–8.04 (0.007)
ANAEETHES-GEMET	–16.09 (0.001)	–4.14 (0.026)

In Table 3, the T-test's degree of freedom of is 2, and the significance level is 0.05. On all testing cases, the *p*-values are all smaller than 0.05, and thus, we can draw the conclusion that ACEA statistically outperforms CEA and HCEA based aligning techniques at the significance level of 5%. It is obvious that through adaptively maintaining PMs, ACEA can effectively explore the whole feasible region and find high-quality alignments.

**Figure 6:**
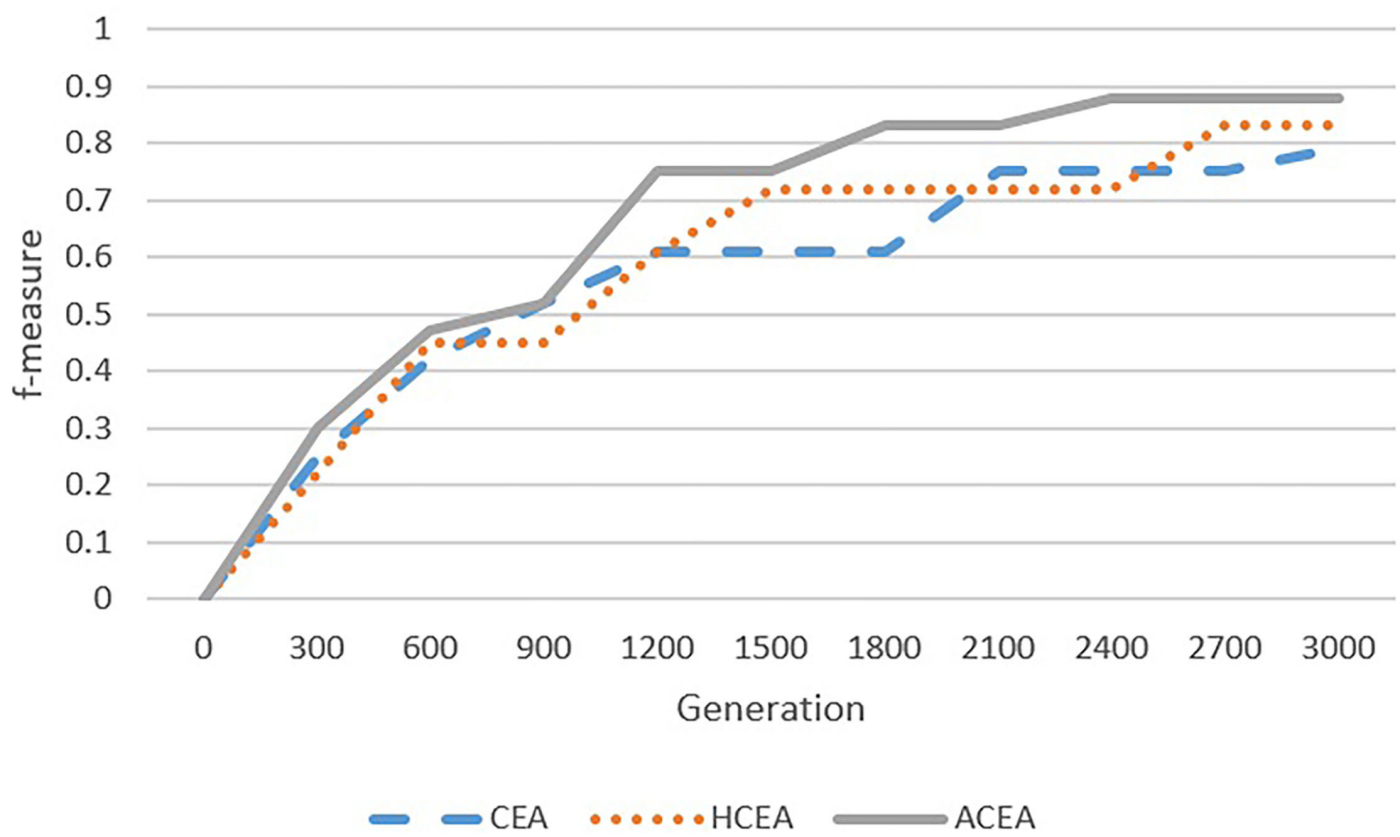
Comparison among ACEA, CEA, and HCEA in terms of convergence graph on ENVO-SWEET.

**Figure 7:**
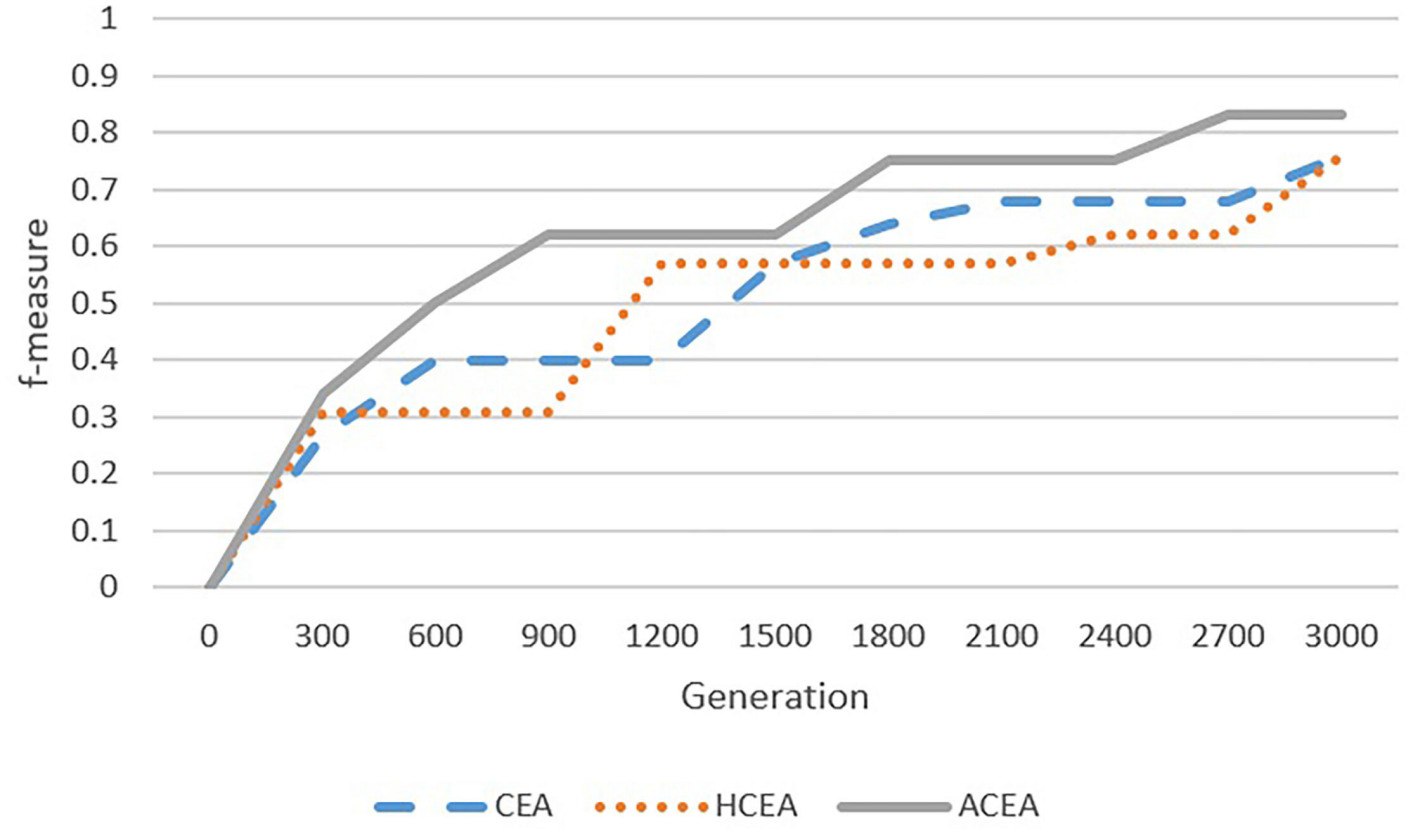
Comparison among ACEA, CEA, and HCEA in terms of convergence graph on FLOPO-PTO.

**Figure 8:**
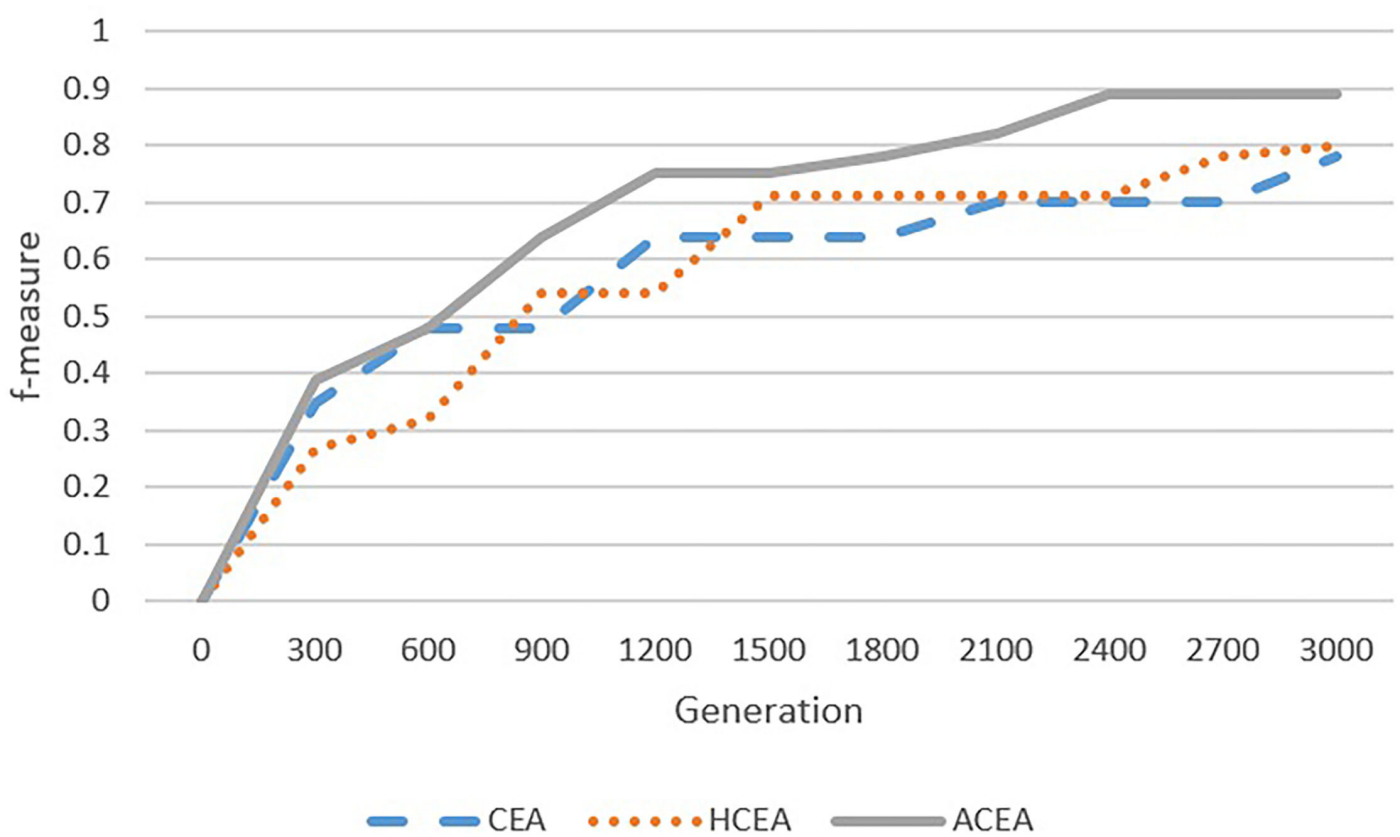
Comparison among ACEA, CEA, and HCEA in terms of convergence graph on AGROVOC-NALT.

**Figure 9:**
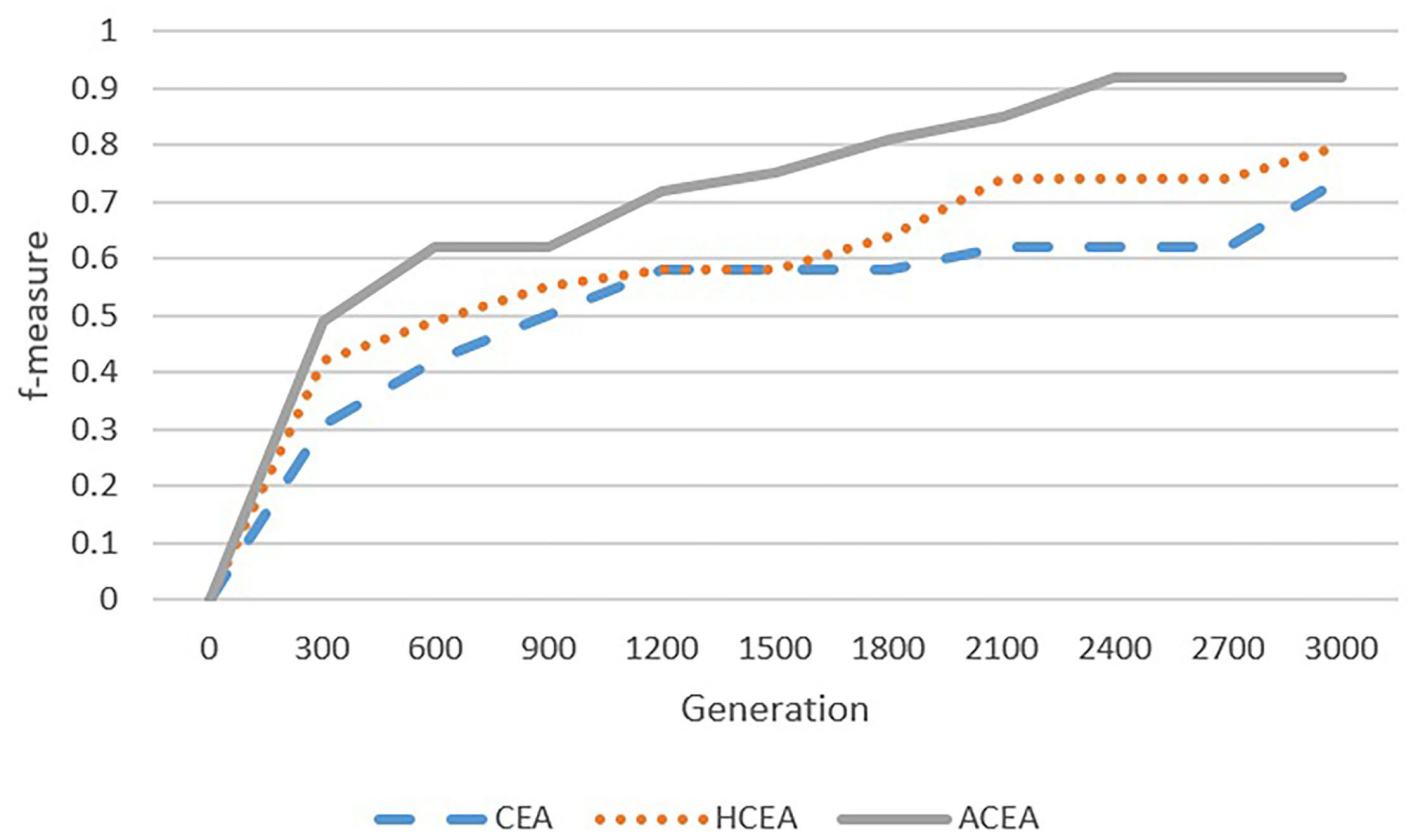
Comparison among ACEA, CEA, and HCEA in terms of convergence graph on ANAEETHES-GEMET.

As depicted in Figures 6–9, with the introduction of semantic reasoning, the searching space of ACEA can be significantly reduced, which makes it able to more efficiently converge to the global optimal solution.

Finally, we compare ACEA with OAEI's participants on Biodiversity and Ecology track through f-measure. In Table 4, we can see that ACEA's mean f-measure outperforms all the state-of-the-art ontology aligning systems on all testing cases. ACEA makes use of the evolutionary paradigm to iteratively refine the alignment's quality, which is a more effective way of improving the alignment's quality than other machine learning based aligning approaches (such as ALOD2Vec, POMap, and DOME), logical reasoning based aligning methods (such as Lily, LogMap Family, and XMap) and Word-based aligning techniques (such as AML, Wiktionary, FCAMapKG, ATBox, and KGMatcher).

**Table 4:** Comparisons among ACEA and state-of-the-art ontology aligning techniques in terms of f-measure.

**Aligning system**	**ENVO-SWEET**	**FLOPO-PTO**	**AGROVOC-NALT**	**ANAEETHES-GEMET**
AML (Lima et al., 2020)	0.84	0.86	0.87	0.85
Lily (Wu et al., 2019)	0.73	0.68	-	-
LogMap (Jiménez-Ruiz, 2020)	0.78	0.80	-	0.89
LogMapBio (Jiménez-Ruiz, 2020)	0.77	0.79	-	0.89
LogMapLite (Jiménez-Ruiz, 2020)	0.77	0.75	-	0.49
POMap (Laadhar et al., 2018)	0.78	0.68	-	-
XMap (Djeddi et al., 2015)	0.78	0.76	-	-
DOME (Hertling and Paulheim, 2018)	-	0.73	-	-
FCAMapKG (Algergawy et al., 2019)	0.63	0.69	-	-
POMap (Laadhar et al., 2018)	0.69	0.68	-	-
ATBox (Hertling and Paulheim, 2020)	0.69	0.71	-	-
Wiktionary (Portisch and Paulheim, 2020)	-	0.002	-	-
ALOD2Vec (Portisch et al., 2020)	-	0.002	-	0.10
KGMatcher (Fallatah et al., 2021)	0.005	-	-	0.063
ACEA	0.88	0.83	0.89	0.92

*The symbol “-” means that the corresponding matching technique is not able to determine an alignment*.

### 5.3. Discussions on Experimental Results

Compact evolutionary algorithm combines the mechanisms of a classic EA with competitive learning, which is effective to lead the algorithm to determine the optimal solution. In addition, the simplicity of CEA, which does not require all the mechanisms of an EA, rather the few steps in the algorithm are small and simple. HCEA further introduces local refinements on the elite solution, which allows increasing the convergence speed *via* the local search. Compared with CEA and HCEA, ACEA works based on the probabilistic modeling of promising solutions, which makes it easier to predict the movements of the populations in the search space. When confronted with complex optimization issues, ACEA is able to jump out of the local optima through adaptively PM maintenance, which guides the algorithm to explore the potential search space and learn a more complex probabilistic model. Therefore, ACEA outperforms CEA and HCEA in terms of both qualities of alignments and computational efficiency.

In addition, ACEA comprehensively aggregates three broad categories of entity similarity measure, i.e., syntactic-based similarity measure, linguistic-based similarity measure, and structure-based similarity measure, which lead to better alignments than the ones that only take into consideration one or two of them, such as AML, LogMap Family, POMap, XMap, DOME, FCAMapKG. This is because when facing a different heterogeneous situation, none of the similarity measures could be effective in all matching tasks, and taking into consideration more similarity measures could be of help to find the correct correspondences.

## 6. Conclusion

To manage knowledge on ecology and biodiversity and preserve the ecosystem and biodiversity simultaneously, it is necessary to link the data entities in different ecology and biodiversity ontologies. To this end, this study proposes an ACEA-based ecology and biodiversity ontology aligning technique. In particular, the problem of EBOA is modeled as a large-scale discrete optimization problem with a sparse solution. Then, a hybrid entity similarity measure is presented to calculate the ecology and biodiversity entities' similarity. Finally, a problem-specific ACEA is proposed, which introduces semantic reasoning and adaptive PM maintenance to efficiently solve the problem of EBOA. The experimental results show that the evolutionary paradigm is able to find a better alignment than other artificial techniques and the proposed semantic reasoning and adaptive PM maintenance are able to further improve the algorithm's efficiency.

Although ACEA based aligning technique shows its superiority in the experiment, it is not able to detect the m:n correspondence, i.e., multiple source entities are mapped with multiple target entities, which is a common complex correspondence pattern. In addition, ACEA is also not able to find other semantic relationships among the entities, such as the subsumption. Finally, the divide-and-conquer approach has been proved to be a viable method that can facilitate the effectiveness of aligning process (Hu et al., 2008), and we are also interested in utilizing the ontology partitioning technique to pre-process two ontologies.

## Data Availability Statement

The raw data supporting the conclusions of this article will be made available by the authors, without undue reservation.

## Author Contributions

XX and P-WT proposed the idea. XX performed the literature review and comparative analyses and wrote the manuscript draft. P-WT performed the experiments and made the figures. All authors read the manuscript draft, commented on it, and confirmed it before submission.

## Funding

This study was supported by the National Natural Science Foundation of China (No. 62172095), the Natural Science Foundation of Fujian Province (No. 2020J01875), and the Scientific Research Foundation of the Fujian University of Technology (No. GY-Z17162).

## Conflict of Interest

The authors declare that the research was conducted in the absence of any commercial or financial relationships that could be construed as a potential conflict of interest.

## Publisher's Note

All claims expressed in this article are solely those of the authors and do not necessarily represent those of their affiliated organizations, or those of the publisher, the editors and the reviewers. Any product that may be evaluated in this article, or claim that may be made by its manufacturer, is not guaranteed or endorsed by the publisher.

## Footnotes

^1^ http://oaei.ontologymatching.org

^2^ http://agroportal.lirmm.fr/ontologies/ENVO

^3^ https://bioportal.bioontology.org/ontologies/SWEET

^4^ http://agroportal.lirmm.fr/ontologies/TO

^5^ http://agroportal.lirmm.fr/ontologies/FLOPO

^6^ http://agroportal.lirmm.fr/ontologies/AGROVOC

^7^ http://agroportal.lirmm.fr/ontologies/NALT

^8^ http://agroportal.lirmm.fr/ontologies/GEMET

^9^ http://agroportal.lirmm.fr/ontologies/ANAEETHES

^10^ http://oaei.ontologymatching.org/2021/biodiv/index.html
